# Host nutrition alters the variance in parasite transmission potential

**DOI:** 10.1098/rsbl.2012.1145

**Published:** 2013-04-23

**Authors:** Pedro F. Vale, Marc Choisy, Tom J. Little

**Affiliations:** 1 CEFE-CNRS–UMR 5175, 1919 Route de Mende, Montpellier 34293, France; 2 MIVEGEC, UMR Universités Montpellier 1 & 2, CNRS 5290, IRD 224, 911 Avenue Agropolis, Montpellier, France; 3 Oxford University Clinical Research Unit, Hanoi, Vietnam; 4 Centre for Immunity, Infection, and Evolution and Institute of Evolutionary Biology, School of Biological Sciences, University of Edinburgh, Ashworth Laboratories, West Mains Road, Edinburgh EH9 3JT, UK

**Keywords:** disease transmission, nutrition, variance, super-shedding, *Daphnia*

## Abstract

The environmental conditions experienced by hosts are known to affect their mean parasite transmission potential. How different conditions may affect the variance of transmission potential has received less attention, but is an important question for disease management, especially if specific ecological contexts are more likely to foster a few extremely infectious hosts. Using the obligate-killing bacterium *Pasteuria ramosa* and its crustacean host *Daphnia magna*, we analysed how host nutrition affected the variance of individual parasite loads, and, therefore, transmission potential. Under low food, individual parasite loads showed similar mean and variance, following a Poisson distribution. By contrast, among well-nourished hosts, parasite loads were right-skewed and overdispersed, following a negative binomial distribution. Abundant food may, therefore, yield individuals causing potentially more transmission than the population average. Measuring both the mean and variance of individual parasite loads in controlled experimental infections may offer a useful way of revealing risk factors for potential highly infectious hosts.

## 1. Introduction

Both the genetic and environmental context are known to affect the mean parasite load (and therefore, the potential for transmission), across a range of host–parasite systems [1]. Few studies however, have explicitly examined how conditions experienced during infection may affect the variance in transmission potential among individual hosts. Individual variation in parasite loads has been well described for macroparasites, with 20 per cent of hosts commonly found carrying approximately 80 per cent of total parasites [2]. The implication of this observation, known as the 20–80 rule, is that disease control can, in principle, achieve an 80 per cent reduction in transmission by identifying and targeting only the 20 per cent most infectious individuals [3–5]. Identifying specific causes of variation in transmission potential among hosts is, therefore, an important step for successful disease control, but is challenging in the context of epidemics owing to the difficulty in quantifying individual infectious loads [6,7].

One alternative is to study infection under a variety of genetic and environmental contexts in controlled experimental conditions, to determine whether some conditions yield greater variance in individual potential infectiousness. Here, we analysed data from a tractable host–parasite model system, the freshwater planktonic crustacean *Daphnia magna* and the obligate-killing bacterial parasite *Pasteuria ramosa,* to study the genetic and environmental influences on the distribution of the lifetime transmission potential of each host. A previous study investigated how the severity of parasitism (measured as the covariance of host and parasite fitness) varied under a range of temperature and food conditions [8]. Given that all hosts were followed until death (when all transmission occurs), here, we present a quantitative analysis of the variance in individual parasite loads under low and high host nutrition levels and test for skewed distributions of transmission potential that reveal highly infectious individual hosts.

## 2. Material and methods

### (a) Experiment

Prior to infection, eight *D. magna* clones were each kept under identical conditions in 12 replicate jars (five *Daphnia* per jar) for at least two generations, to minimize maternal effects on the experimental generation (see [8] for details). Female offspring from each of the 12 replicate jars were then placed individually in jars containing artificial pond medium and split into different food and temperature treatments: high food−absorbance_λ__=__665nm_ = 1.5, low food absorbance_λ__=__665nm_ = 0.3 of chemostat-grown *Chlorella vulgaris* microalgae, at 15°C, 20°C and 25°C. We exposed 5-day-old female *Daphnia* individually to *P. ramosa* for 5 days. Hosts were then transferred to clean medium and observed daily for signs of infection, evident by an empty brood chamber and altered coloration 20 days post-exposure. *Daphnia* that died during infection were stored at −20°C. We quantified *P. ramosa* spores by crushing *Daphnia* and counting spores using a CASY Cell Counter Model TT (Schärfe System GmbH, Reutlingen, Germany).

### (b) Analysis

When the number of parasites is randomly distributed among hosts, the distribution of parasite loads is expected to follow a Poisson distribution with a variance equal to its mean: *σ*^2^ = *μ*. A simple way to account for sources of heterogeneity among hosts is to consider a mixture of Poisson distributions with different means, for example with the commonly used negative binomial distribution. The variance *σ*^2^ of a negative binomial depends on both the mean *μ* and the dispersion parameter *k*:
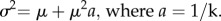


When *a* is close to 0 (in practice *k* > 20), the negative binomial distribution reduces to a Poisson, and we can see from the above equation that the variance equals the mean again. For this reason, the sample variance-to-mean ratio (*σ*^2^/*μ*, also called the index of dispersion) is classically used as a measure of heterogeneity (how long the ‘tail’ of the distribution is): it varies from zero for uniformly distributed parasites (zero variance), takes a value of 1 when the number of parasites is randomly distributed (Poisson distribution), or is higher than unity when parasites are aggregated (negative binomial distribution; [2,9]). We used the maximum-likelihood method to estimate the mean *μ* and dispersion parameter *k* of negative binomial distributions on our experimentally measured spore counts for low and high food treatments [10]. Significance of heterogeneities and aggregation (*H*_0_: *a* = 0 or *σ*^2^/*μ* > 1) was tested by likelihood ratio tests between negative binomial and Poisson distributions. We further calculated the goodness of fit of each distribution as the squared correlation coefficient *r*^2^ between the observed and predicted cumulative distribution functions. Within each food treatment we further tested whether host genotype or temperature treatments affected the distributions significantly using a tree-based modelling approach (see the electronic supplementary material). Analyses of the data were carried out in R (R Development Core Team, www.R-project.org). The raw data used in the analyses may be found in the Dryad data depository (http://dx.doi.org/10.5061/dryad.f1338).

## 3. Results

We exposed 541 individual *Daphnia* to *P. ramosa* and 229 (42%) developed infection. The probability of becoming infected varied between host genotypes (*χ*^2^_7_ = 65.93, *p* < 0.001).

**Figure 1.**
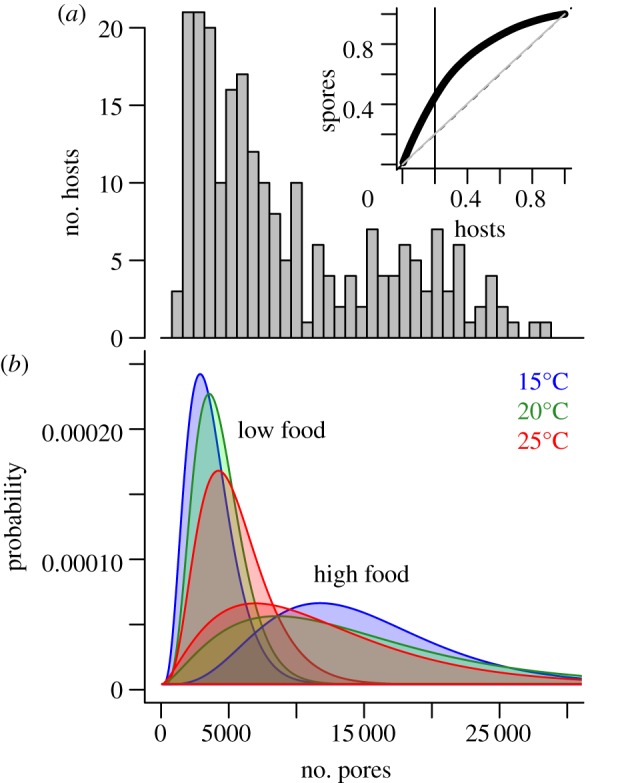
(*a*) Distribution of individual parasite loads across all hosts. Inset: the total transmission spores released by hosts, ranked by their infectiousness. The vertical black line indicates the fraction of total transmission caused by the 20% most infectious hosts under homogeneous transmission (dashed line), under a Poisson distribution with the same mean (full line), or under the actual over-dispersed data shown in (*a*). Note that dashed and full lines overlap. (*b*) Estimated probability distributions for the low and high food and temperature treatments. Detailed analysis in the electronic supplementary material.

Under limiting food, the mean parasite load of each host was lower (*F*_2195_ = 525.03, *p* < 0.001; figure 1) and the variance-to-mean ratio was closer to 1 (table 1). A negative binomial distribution did not fit the data better than a Poisson at low food (*χ*^2^_1_ = 1.0781, *p* = 0.2991; table 1). By contrast, well-fed hosts produced on average more transmission-stage spores per individual (table 1 and figure 1), and the distribution of individual parasite loads showed evidence of higher aggregation relative to poorly fed hosts (shown by the higher variance-to-mean ratio; table 1). Accordingly, individual parasite loads under abundant food were well described by a negative binomial distribution (*r*^2^ = 0.991; table 1), which fit the data significantly better than a Poisson distribution (*χ*^2^_1_ = 160.04, *p* < 0.0001). The potential consequences of this aggregation are apparent when ranking hosts by their contribution to the total transmission: the most infectious 20 per cent of hosts produced approximately 45% of the total parasite transmission spores (figure 1*a*, inset). While not as extreme as the 20–80 rule, this more than double the expectation under a homogeneous transmission distribution.

**Table 1.** Summary statistics and maximum-likelihood model fits of the distribution of individual infectiousness potential. (*n*, number of hosts; *μ*, mean number (×10^5^) of parasite spores per hosts on the day of death; *s^2^*, variance; *s*^2^/*m*, variance to mean ratio; *k*, maximum-likelihood estimate of negative binomial dispersion parameter; *λ*, maximum-likelihood estimation of Poisson distribution parameter; s.e. are standard errors of the estimated parameters; LL, log-likelihood of distribution fits to the data; *Δ*LL, difference in log-likelihood of the two model fits; *p*, probability that there is no difference between model fits.)

	summary statistics					negative binomial distribution			Poisson distribution			*Δ*LL	
	*n*	mean (×10^5^ spores) *m*	s.e.m.	variance, *s*^2^	*s*^2^/*m*	*k*	s.e. (*k*)	LL	*λ*	s.e. (*λ*)	LL		*p*
food level													
low	111	4.59	0.22	5.01	1.08	31.9	33.75	−243.99	4.59	0.2	−244.53	−1.08	0.30
high	118	13.94	0.7	47.66	3.42	4.49	0.8	−397.61	13.94	0.34	−477.63	−160.04	<0.0001

**Figure 2.**
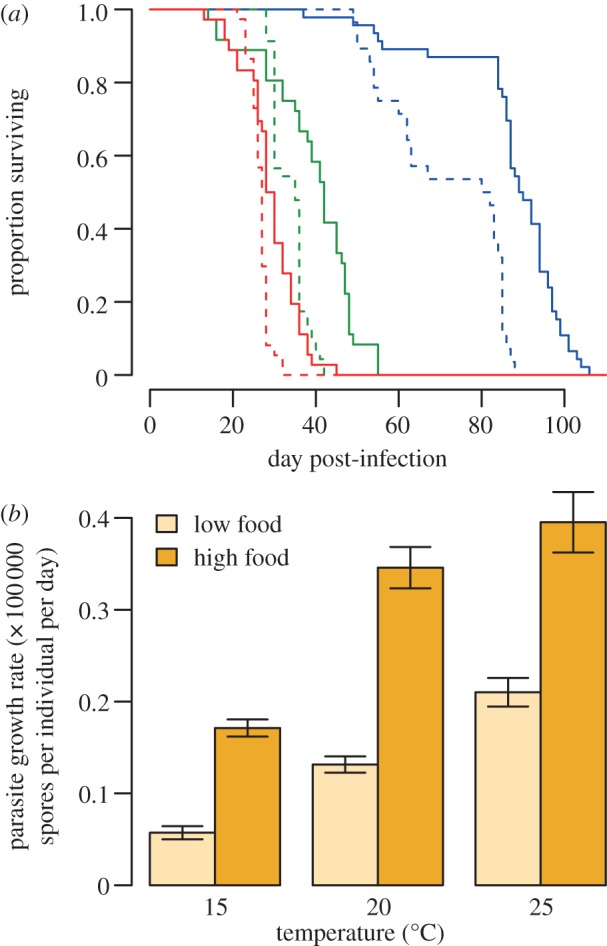
(*a*). Host survival under high (full line) or low (dashed line) food at 15°C (blue), 20°C (green) and 25°C (red). (*b*). Parasite growth rate (the number of parasite spores per individual per day alive) when infecting hosts were kept under low (pale yellow) or high food (dark yellow). Error bars are s.e.m.

Within each food treatment, fitting separate distributions for each temperature treatment significantly improved the estimates of *μ* and *k* (table 1 and figure 1), while host genotype effects were not detected (see the electronic supplementary material, for detailed analysis). Abundant food resulted in higher host survival (*F*_2,195_ = 1611.75, *p* < 0.001; figure 2) and parasite within-host growth rates approximately doubled compared with conditions of limiting host nutrition (figure 2). Host genotype did not influence the timing of host death (*F*_7,195_ = 1.54, *p* = 0.16).

## 4. Discussion

Why do some hosts transmit more? The mean parasite load per individual host is known to vary with the environmental conditions experienced during infection in many host–pathogen systems [1], but few studies have explicitly examined how conditions may alter the distribution of individual parasite loads. The distinction is important, because the mean parasite load may conceal the presence of a few extremely infectious individuals at the tail of the distribution that accelerate disease spread [6].

We examined how the nutritional state of hosts may affect the variance in transmission potential. Not only did poorly fed hosts contribute relatively few spores, we found little variation around the mean transmission. Under abundant food however, the variance was much higher and individual parasite loads followed a negative binomial distribution where a few hosts showed high transmission potential (figure 1). These individual hosts could arise if increased host nutrition allows elevated parasite growth rates—for example, owing to a greater allocation of host resources to parasite growth. Indeed, we found that parasite within-host growth rates were higher in well-fed hosts (figure 2). This more aggressive exploitation of host resources could be expected to result in a greater rate of host mortality, but we also observed that well-fed hosts were generally longer-lived than those in the lower food treatment, and this allowed parasites more time to grow within hosts (figure 2).

Well-fed hosts that lived longer also had higher mean parasite loads (see figure 1 in [8]), but it is unclear why high food would also result in increased variance in transmission potential. One possibility, given that all transmission happens at host death, is that abundant food, alters the survival distribution of hosts, indirectly affecting variance in parasite loads. Interestingly, we observed relatively higher variance in the more stressful temperature (25°C, electronic supplementary material, table S1). This is consistent with Schmalhausen's law, which states that organisms experiencing stressful conditions will show greater variance in their life-history traits [11].

Host nutrition is likely to be a key factor in how epidemics progress, because immune defences are energetically costly and rely heavily on host resource intake [12]. Hall *et al*. [13] examined the effect of resource quality on the progression of a fungal disease in the planktonic crustacean *Daphnia dentifera* in both natural and laboratory infections. They found that poor food quality may be responsible for delaying the start of epidemics, owing to reduced parasite growth rates in inadequately nourished hosts. This is consistent with our result showing that under low food the mean and variance in individual parasite loads are low. More generally, conditions that allow high parasite loads with a reduced effect on host health could result in poorer detection of infection, compromising disease control efforts [14].

The transmission potential of individuals is only one component of disease spread, and the contact network of infected individuals [15] and genotype-specific differences in the likelihood to carry and transmit infection [16] will certainly also play a role. Our study included eight genotypes of *Daphnia* and, as previously reported [17], we found host genotype effects on the susceptibility to infection, implying that individual variation in transmission potential is affected by the environment (in this case, food level), whereas variation in the initial susceptibility to infection has a stronger genetic component [18]. Ultimately, successful control of infection will require empirical measurements of both individual transmission potential and susceptibility for multiple host genotypes under commonly experienced environmental variation [7].
